# Integrative ATAC-seq and RNA-seq analyses of IPEC-J2 cells reveals porcine transcription and chromatin accessibility changes associated with *Escherichia coli* F18ac inhibited by *Lactobacillus reuteri*

**DOI:** 10.3389/fmicb.2023.1101111

**Published:** 2023-02-16

**Authors:** Weiyun Qin, Yunxiao Xie, Zhanshi Ren, Chao Xu, Ming-an Sun, Zongjun Yin, Wenbin Bao

**Affiliations:** ^1^College of Animal Science and Technology, Yangzhou University, Yangzhou, China; ^2^College of Veterinary Medicine, Institute of Comparative Medicine, Yangzhou University, Yangzhou, China; ^3^College of Animal Science and Technology, Anhui Agricultural University, Hefei, Anhui, China

**Keywords:** diarrhea, *Lactobacillus reuteri*, *Escherichia coli* F18ac, chromatin accessibility, transcriptome

## Abstract

*Escherichia coli* is the main cause of postweaning diarrhea in pigs, leading to economic loss. As a probiotic, *Lactobacillus reuteri* has been used to inhibit *E. coli* in clinical applications; however, its integrative interactions with hosts remain unclear, especially in pigs. Here, we found that *L. reuteri* effectively inhibited *E. coli* F18ac adhering to porcine IPEC-J2 cells, and explored the genome-wide transcription and chromatin accessibility landscapes of IPEC-J2 cells by RNA-seq and ATAC-seq. The results showed that some key signal transduction pathways, such as PI3K-AKT and MAPK signaling pathways, were enriched in the differentially expressed genes (DEGs) between *E. coli* F18ac treatment with and without *L. reuteri* groups. However, we found less overlap between RNA-seq and ATAC-seq datasets; we speculated that this might be caused by histones modification through ChIP-qPCR detection. Furthermore, we identified the regulation of the actin cytoskeleton pathway and a number of candidate genes (*ARHGEF12*, *EGFR*, and *DIAPH3*) that might be associated with the inhibition of *E. coli* F18ac adherence to IPEC-J2 cells by *L. reuteri*. In conclusion, we provide a valuable dataset that can be used to seek potential porcine molecular markers of *E. coli* F18ac pathogenesis and *L. reuteri* antibacterial activity, and to guide the antibacterial application of *L. reuteri*.

## 1. Introduction

Bacterial diarrhea is one of the most serious causes of postweaning diarrhea (PWD), which endangers the sustainable development of the pig industry in China and is especially harmful to the health of piglets (Wu et al., 2016). Bacterial diarrhea is mainly caused by pathogenic *Escherichia coli* F18. Several of its common clinical symptoms include diarrhea, decreased growth velocity, weight loss, and death (Moxley and Duhamel, 1999). Porcine pathogenic *E. coli* strains harbor specific colonization factors, including fimbrial adhesins F18 and F4 (K88; Fairbrother et al., 2005). There are 2 closely related antigenic variants of F18, F18ab, and F18ac. While F18ab-positive strains are known to be associated with edema disease, *E. col*i-carrying F18ac are known to cause diarrhea (DebRoy et al., 2009). The development of resistance to widely used antibiotics in a variety of *E. coli*, as well as the increased prevalence and gravity of postweaning syndrome, urgently require the use of alternative strategies to control them (Fairbrother et al., 2005). Of the probiotics and postbiotics used as substitutes for antibiotics, of particular interest are the bacteriocinogenic probiotics, that is, bacterial strains capable of producing bacteriocins that confer health benefits to the host.

*Lactobacillus reuteri* is a gram-positive bacterium belong to *Firmicutes*, *Bacilli*, *Lactobacillales*, *Lactobacillaceae*, *Limosilactobacillus* (Zheng et al., 2020), which can colonize in gastrointestinal tract and then has a variety of beneficial effects on diarrhea, intestinal infection, inflammatory bowel syndrome (IBS), inflammatory bowel disease (IBD), and colorectal cancer (Lebeer et al., 2008; Dore et al., 2014; Mu et al., 2018; Dore et al., 2019). *L. reuteri* may be a useful, safe, and supportive measure for the treatment and prevention of diarrhea, reducing both the duration and the intensity of diarrhea symptoms, having beneficial health effects (Szajewska et al., 2014). *L. reuteri* ATCC 53608, isolated from pig intestines also has a similar antibacterial effect, which against pathogenic (*Staphylococcus aureus Salmonella enterica* ssp. *enterica*, and *Listeria monocytogenes*), and pathogen surrogate (*Escherichia coli* DH5α) microorganisms (Ortiz-Rivera et al., 2017). However, our understanding of its specific intestinal protection and antibacterial mechanisms is still limited. Notably, oral administration of *L. reuteri* to healthy breastfed mice promoted intestinal immune tolerance and was linked to the proliferation of beneficial gut microbiota (Liu et al., 2019). *L. reuteri* also induced an anti-inflammatory response by affecting the secretion of macrophage-derived cytokines (Dias et al., 2021). Additionally, *L. reuteri*, together with a tryptophan-rich diet, could reprogram intraepithelial CD4(+) T cells into immunoregulatory T cells (Cervantes-Barragan et al., 2017). *L. reuteri* protected the intestinal mucosal barrier integrity by moderately modulating the Wnt/β-catenin pathway to avoid overactivation (Wu et al., 2020). The understanding of these mechanisms and of regulatory changes at the genomic chromatin level remains incomplete.

With the popularization of high-throughput sequencing, revealing changes in host genome-wide chromatin has become possible using sequencing; as a tool, accessible chromatin with next-generation sequencing (ATAC-seq) can be used to detect the unique chromatin landscape associated with a cell type and how it may be altered by perturbation or disease (Grandi et al., 2022). The advantage of ATAC-seq is that it requires only a small number of viable cells and does not require knowledge about transcription factors or epigenetics. In this study, ATAC-seq helped us to actively understand the epigenetic changes that regulate the host during the pathogenic process of *E. coli* F18ac, and how *L. reuei* inhibits *E. coli* F18ac to protect the host cells. We aimed provide unique insights into further understanding of the mechanisms of bacterial interaction with the host at the chromatin level by using ATAC-seq combined with RNA-seq. Our findings also provide a strong reference and basis for the efficient use of the antibacterial properties of *L. reuteri*.

## 2. Materials and methods

### 2.1. Bacterial culture

We purchased *L. reuteri* (ATCC 53608) from the China Center of Industrial Culture Collection (CICC) (Beijing, China), which we grew in de Man, Rogosa, and Sharpe (MRS) medium at 37°C for 24 h under anaerobic conditions. *E. coli* F18ac was offered by the veterinary laboratory at the University of Pennsylvania, and was inoculated in Luria-Bertani (LB) medium at 37°C for 24 h. For subsequent cell assays, we collected bacterial cells by centrifugation at 4000 *r*/min for 5 min and washed with phosphate-buffered saline (PBS) buffer three times. Finally, we used Dulbecco’s modified eagle medium (DMEM) with 10% fetal bovine serum to resuspend the bacterial cells, which we adjusted to a density of 1 × 10^7^ colony forming units (CFU)/mL *E. coli* F18ac and 1 × 10^8^ CFU/ml of *L. reuteri*.

### 2.2. Zone of inhibition assay

We evenly distributed *E. coli* F18ac over the surface of the LB plate. After the plate was dried, 200 μL of *L. reuteri* fermentation broth was added into each Oxford cup before the pH was adjusted to 6.0 using NaOH solution, which we transferred to a 37°C incubator for overnight. We compared the inhibition zone diameter values with the prescribed Kirby-Bauer antibiotic testing standard values to determine whether they were resistive, susceptible, or intermediate (Cockerill, 2010).

### 2.3. Assay of adhesion ability of bacteria to IPEC-J2 cells

We seeded IPEC-J2 cells at a density of 0.7 × 10^6^ cells/mL with DMEM, which we then supplemented with 10% fetal bovine serum. We grew cells to complete confluence, which we subsequently washed three times with PBS and incubated for 4 h in DMEM with *E. coli* F18ac or simultaneously added extra *L. reuteri* (Skjolaas et al., 2007; Liu et al., 2017; Yi et al., 2018). For the control group, we replaced the medium only. We washed unadhered bacteria with PBS five times; then, we digested the culture cells with trypsin. We used colony counting to calculate the number of bacteria.

### 2.4. Sample collection

**Figure 1:**
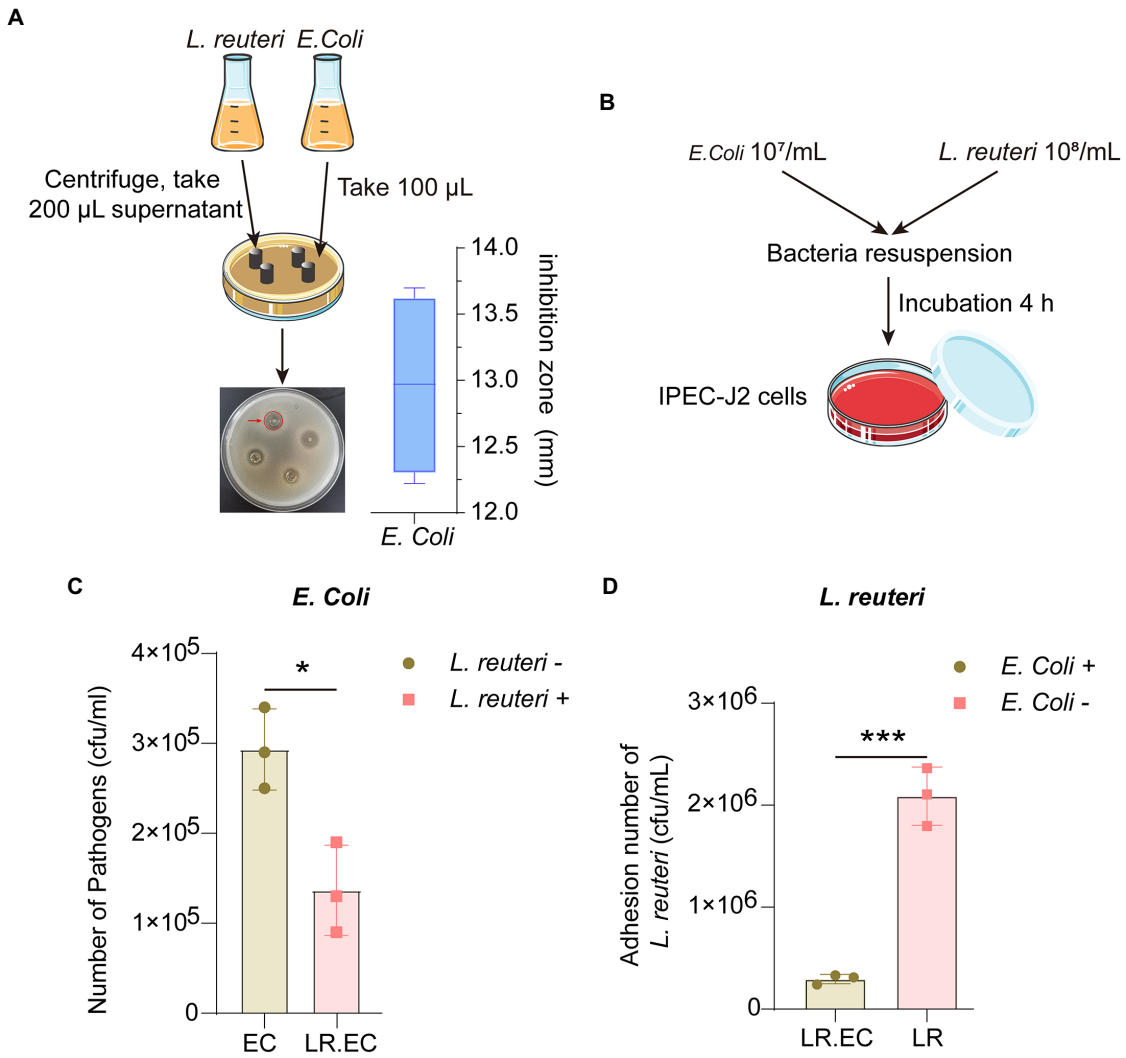
Antibacterial effect of *L. reuteri in vitro*. **(A)** Zone diameters of inhibition of *L. reuteri* against *E. coli* F18ac. **(B)** Workflow of adhesion ability assay of bacteria to IPEC-J2 cells. **(C)** Effect of *L. reuteri* on *E. coli* F18ac adhesion to IPEC-J2 cells. **(D)** Adhesion of *L. reuteri* to IPEC-J2 cells in *E. coli* F18ac (vs. control). *p < 0.01; ***p < 0.001.

Figure 1 shows our sample treatment and sequencing process. We divided 18 samples into three 3: control (CTL) group, *E. coli* F18ac (EC), *L. reuteri* and *E. coli* F18ac (LR + EC) groups. IPEC-J2 cells were seeded into 25 cm^2^ cell culture flask at a density of 0.7 × 10^6^ cells/mL. Cells were maintained with DMEM supplemented with 10% fetal bovine serum in a cell incubator (37°C, under 5% CO_2_). When the cells were grown to complete confluence, they were incubated for 4 h in DMEM with *E. coli* F18ac or extra *L. reuteri* were simultaneously added, without antibiotics (Skjolaas et al., 2007; Liu et al., 2017; Yi et al., 2018). In the control group, only the medium was replaced. We collected three replicate samples for ATAC-seq and RNA-seq from each group. We subsequently IPEC-J2 cells collected for high-throughput sequencing.

### 2.5. RNA-seq library construction

We performed RNA-seq using the following method. Briefly, we generated the libraries with 3 μg of RNA per sample. We used an NEBNext^®^ UltraTM RNA Library Prep Kit for Illumina® (NEB, United States) following the manufacturer’s protocols. We used an AMPure XP system (Beckman Coulter, Beverly, United States) to purify the fragments, from which we selected lengths 250 and 300 bp. Then, we digested cDNA with USER Enzyme (NEB, United States), and subsequently performed PCR. We purified the PCR products and clustered the index-coded samples on an Agilent Bioanalyzer 2,100 system (Agilent Technologies, CA, United States) and a TruSeq PE Cluster Kit v3-cBot-HS (Illumina, CA, United States), respectively. Finally, we performed RNA-seq on an Illumina HiSeq platform according to standardized procedures.

### 2.6. RNA-seq data processing

We used Trimmomatic to process raw data, including cutting adapter and trimming low-quality bases. We calculated the Q20, Q30, and GC contents. All the subsequent analyses were based on the high-quality data after processing. We obtained the porcine reference genome and all gene annotation data from the Genome website. We built an index of the reference genome, and we aligned paired-end clean reads to the reference genome using Hisat2 (version 2.0.5). We counted the read numbers mapped to each gene. The threshold was set as follows: FDR < 0.05, |fold change| ≥ 2.

### 2.7. Gene ontology and Kyoto encyclopedia of genes and genomes enrichment analysis

We explored Gene ontology (GO) terms and Kyoto encyclopedia of genes and genomes (KEGG) pathways enriched by the differentially expressed genes (DEGs) or annotated genes from differentially accessible regions (DARs) through GO and KEGG analyses. GO and KEGG enrichment analyses are widely used to reflect the relationship between genes and GO terms and pathways. We calculated the significance level as previously described (Chen et al., 2016).

### 2.8. ATAC-seq library construction

We performed ATAC-seq for IPEC-J2 cells according to Buenrostro’s method (Buenrostro et al., 2013), Briefly, we lysed cells with cold lysis buffer 10 mM tris–HCl (pH 7.4), 10 mM NaCl, 3 mM MgCl2, and 0.1% Tween 20. We enriched DNA sequences in open chromatin regions with Tn5 transcriptase. We suspended the cell nucleus with a Tn5 transcriptase reaction system, and we purified the DNA at 37°C for 30 min. Then, we performed the PCR amplification reaction. We obtained cleaned up libraries through PCR amplification reactions and then conducted onboard sequencing with the Illumina platform.

### 2.9. ATAC-seq data analysis

We trimmed the reads in FASTQ format and aligned them to the reference genome of *sus scrofa* (Sscrofa11.1, INSDC Assembly: GCA_000003025.6) using the Bowtie2 software (Langmead and Salzberg, 2012). We used DeepTools (version 2.07; Ramirez et al., 2014) to map the density distribution of each gene. We used MACS2 (version 2.1.1; Zhang et al., 2008) for peak calling extraction. We estimated the empirical false detection rate (FDR), and selected FDR < 0.05 we as the identified peak. We used DESeq2 software (Love et al., 2014) for differential screening of the samples in each group. We used the ChIPseeker package (Yu et al., 2015) for the functional annotation of genome-wide peaks.

### 2.10. ChIP-qPCR

We fixed IPEC-J2 cells with 1% formaldehyde for 10 min, which we then quenched with 2.5 M glycine for 5 min, and sonicated to fragments of 200–500 bp in length. Subsequently, we incubated the chromatin fragments with anti-H3K4me3 and anti-H3K27ac, sequentially, which we then reverse-crosslinked. We purified ChIP-DNA for qPCR; the primer details are listed in Supplementary Table S1.

### 2.11. Indirect immunofluorescence assay

Cells were prepared using 24-well culture plates and fixed in 4% paraformaldehyde, blocked with 5% BSA for 2 h after 0.05% Triton X-100 treatment, and incubated 1 h with phalloidin-FITC (1:1000, Abcam). After washing, the cells were stained with DAPI (1,800, Beyotime Biotechnology), and observed and photographed with a fluorescence microscope.

### 2.12. Statistical analysis and data availability

All the replicates in each group are presented as the mean ± SD. A two-sided Student’s *t*-test was used to analyze the differences between two groups. Standard analysis of variance (ANOVA) was used to analyze the differences among three groups. We considered differences as significant at * *p* < 0.05, ** *p* < 0.01, and *** *p* < 0.001.

## 3. Results

### 3.1. Effective inhibition of *Escherichia coli* F18ac by *Lactobacillus reuteri in vitro*

Because *L. reuteri* can effectively inhibit *E. coli in vitro*, we first investigated the efficiency of *L. reuteri* inhibition of *E. coli* F18ac *in vitro*. The Oxford cup results showed that the inhibition zone diameter reached 12.97 ± 0.69 mm, which is considered intermediate inhibition (Figure 1A). More importantly, *L. reuteri* acts as a probiotic that inhibits bacteria in the intestine and regulates host cell activity. As an effective intestinal epithelial cell model (Peterson and Artis, 2014), we used IPEC-J2 cells to perform bacterial adhesion assays (Figure 1B), effectively revealing that *L. reuteri* significantly reduced the adhesion level of *E. coli* F18ac to IPEC-J2 cells by *in vitro* occupancy (*p* < 0.05). In contrast, the adhesion level of *L. reuteri* also decreased (*p* < 0.001; Figures 1C,D). Our results indicated that *L. reuteri* effectively inhibited *E. coli* F18ac in IPEC-J2 cells.

### 3.2. RNA-seq reveals pathways regulated during bacteriostatic process of *Lactobacillus reuteri*

**Figure 2:**
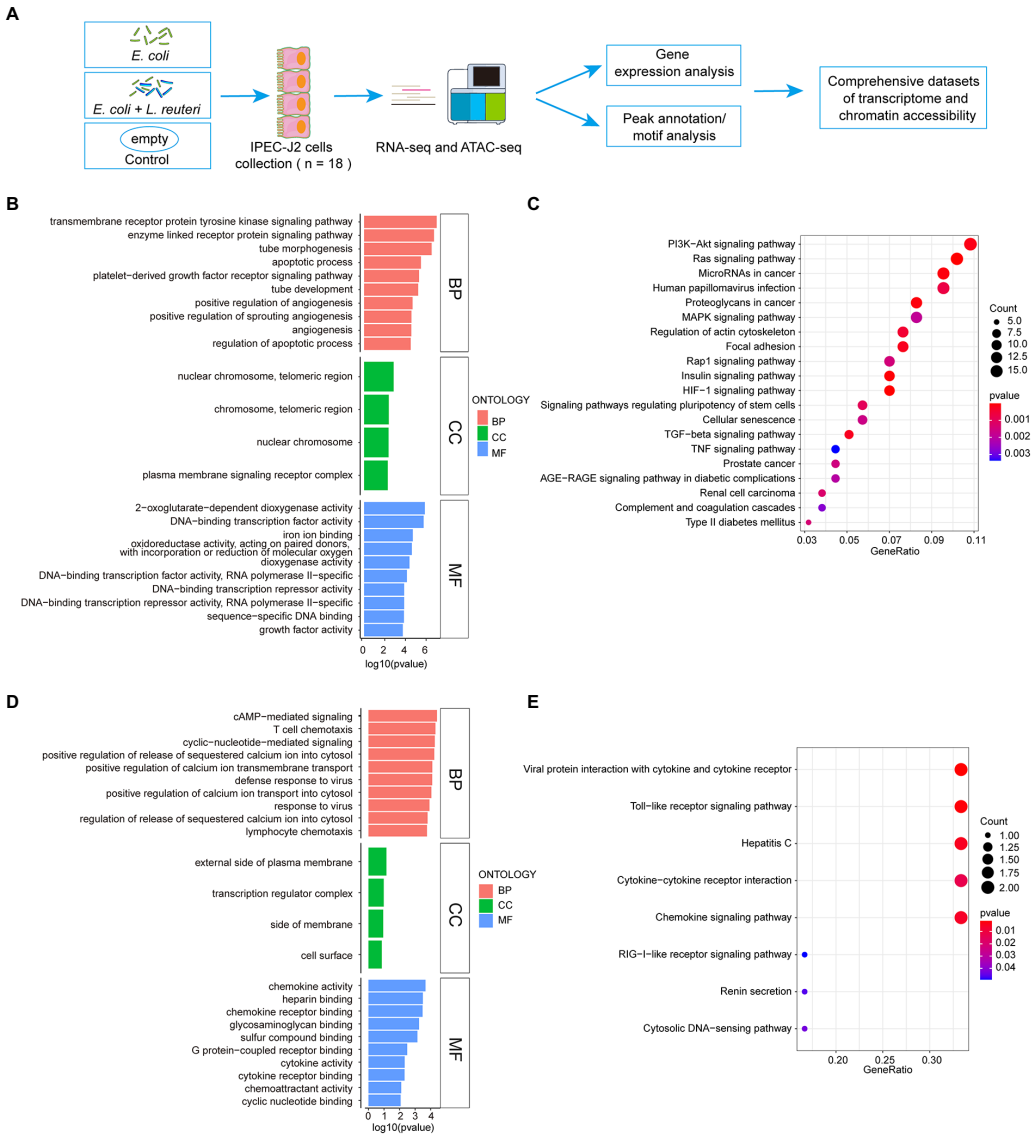
Workflow of sequence and enrichment analyses of RNA-seq dataset. **(A)** Workflow of experimental procedure and sequence analysis. **(B,C)** GO and KEGG enrichment analyses between *E. coli* F18ac and control groups. **(D,E)** GO and KEGG enrichment analyses between *E. coli* F18ac with and without *L. reuteri* groups. BP, Biological Process; CC, Cell Component; MF, Molecular Function.

**Figure 3:**
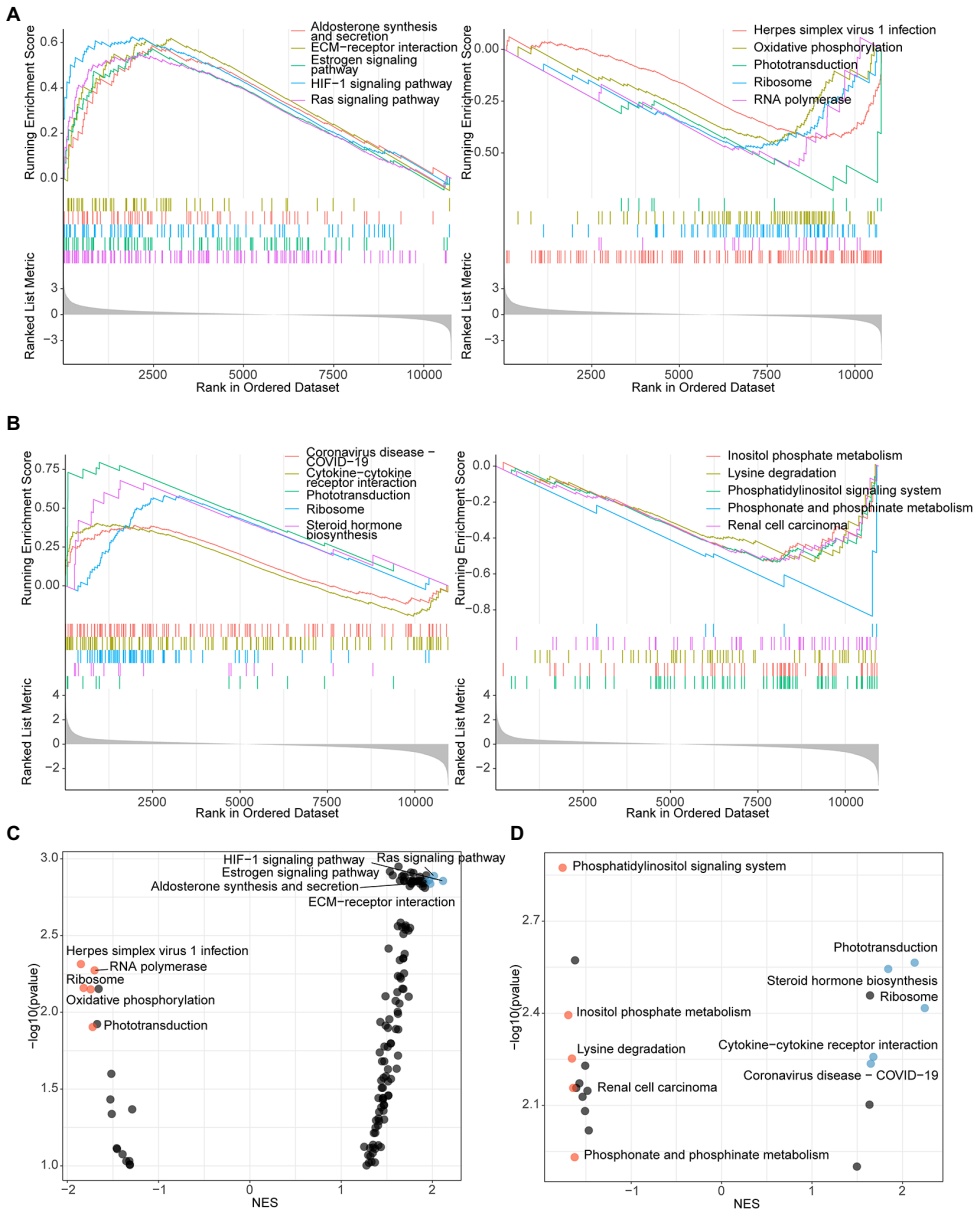
GSEA analysis identified pathways that participate in antibacterial process of *L. reuteri*. **(A)** Up- and downregulated enriched genes of top 5 pathways between *E. coli* F18ac and control groups. **(B)** Up- and downregulated enriched genes of top 5 pathways between *E. coli* F18ac with and without *L. reuteri* groups. Dot plots showing the up- and downregulated pathways in descending order on the y-axis by NES: left plot presents pathways compared between *E. coli* F18ac and control groups **(C)**; right plot represents pathways compared between *E. coli* F18ac with and without *L. reuteri* groups **(D)**. Blue and yellow dots indicate top 5 upregulated and downregulated pathways, respectively.

To thoroughly investigate the regulatory mechanism of the antibacterial activity of *L. reuteri*, we performed RNA-seq and ATAC-seq on IPEC-J2 cells with different treatments (Figure 2A). After quality control, we assessed the high-quality raw data (Supplementary Table S2) prior to downstream analysis. The results of PCA showed that each group could be well separated except for LR.EC sample 2 (Supplementary Figures S1A,D). The results of GO and KEGG enrichment analyses showed that the 373 DEGs in the *E. coli* F18a group compared with the control group were most enriched in the transmembrane receptor protein tyrosine kinase signaling pathway (Figure 2B) and the HIF-1 signaling pathway (Figure 2C). We screened only seven DEGs in *E. coli* F18a and *L. reuteri* co-incubation group compared with the *E. coli* F18a group; they were most enriched in cAMP-mediated signaling (Figure 2D) and viral protein interaction with cytokine and cytokine receptor (Figure 2E). Because a few DEGs were enriched after *L. reuteri* treatment, we performed GSEA to find pathways that play critical roles in the antibacterial process of *L. reuteri*. We found 97 upregulated pathways and 11 downregulated pathways between *E. coli* F18ac and the control groups (Figures 3A,C; Supplementary Table S3), and 8 upregulated pathways and 13 downregulated pathways between *E. coli* F18ac with or without *L. reuteri* groups (Figures 3B,D; Supplementary Table S4). Among these, we found 12 pathways that were upregulated after *E. coli* F18ac treatment, then were downregulated after *L. reuteri* treatment, and 4 pathways that were downregulated after *E. coli* F18ac treatment and then upregulated after *L. reuteri* treatment by examining the intersection (Supplementary Figure S2). These pathways may play an important role in the antibacterial process of *L. reuteri*.

### 3.3. Identification of accessible chromatin in IPEC-J2 cells using ATAC-Seq analysis

**Figure 4:**
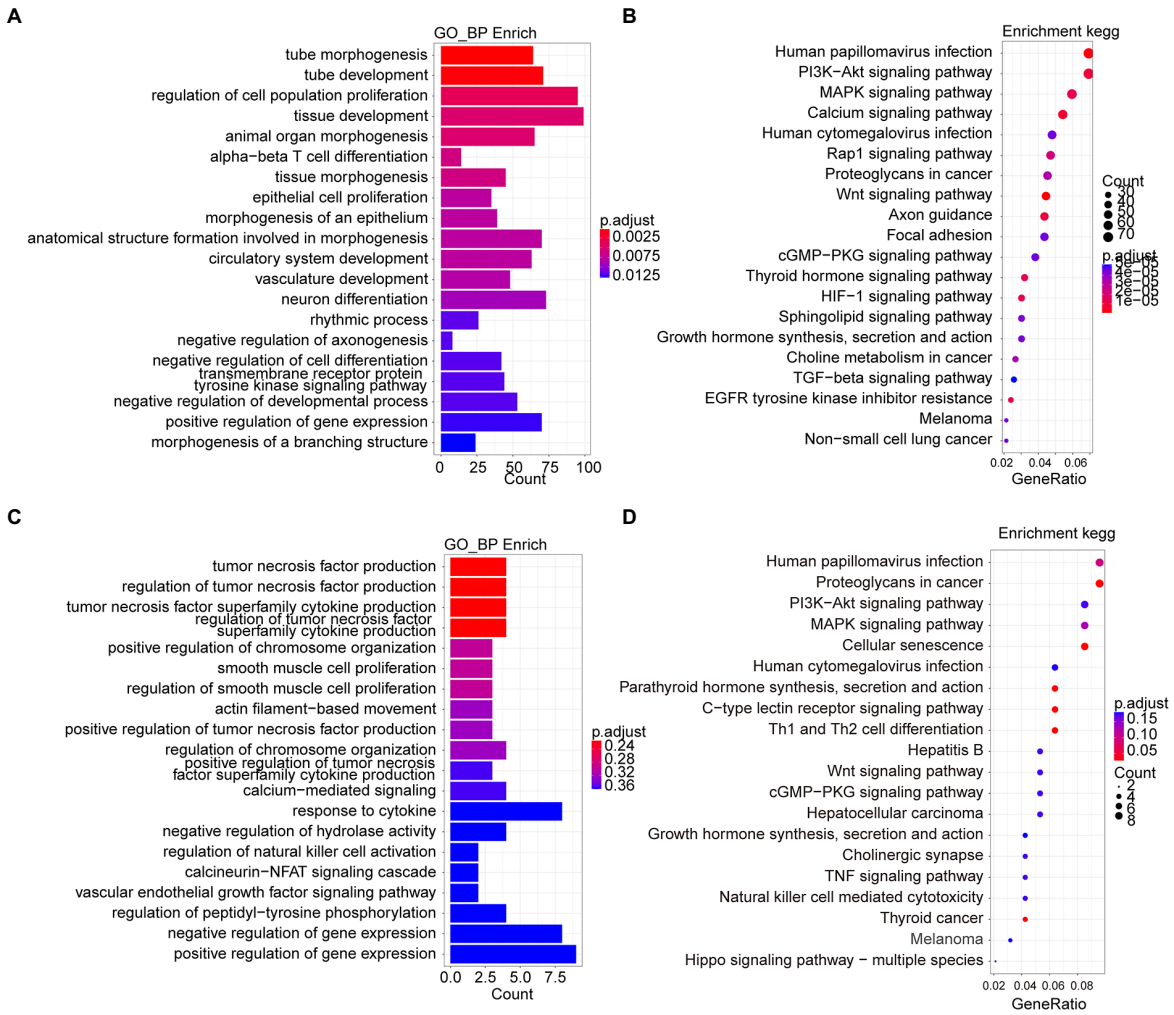
Go and KEGG enrichment analyses from ATAC-seq dataset reveal mechanism of antibacterial process of *L. reuteri*. **(A,B)** GO and KEGG enrichment analyses between *E. coli* F18ac and control groups. **(C,D)** GO and KEGG enrichment analyses between *E. coli* F18ac with and without *L. reuteri* groups. Enriched genes were annotated by DARs from ATAC-seq.

**Figure 5:**
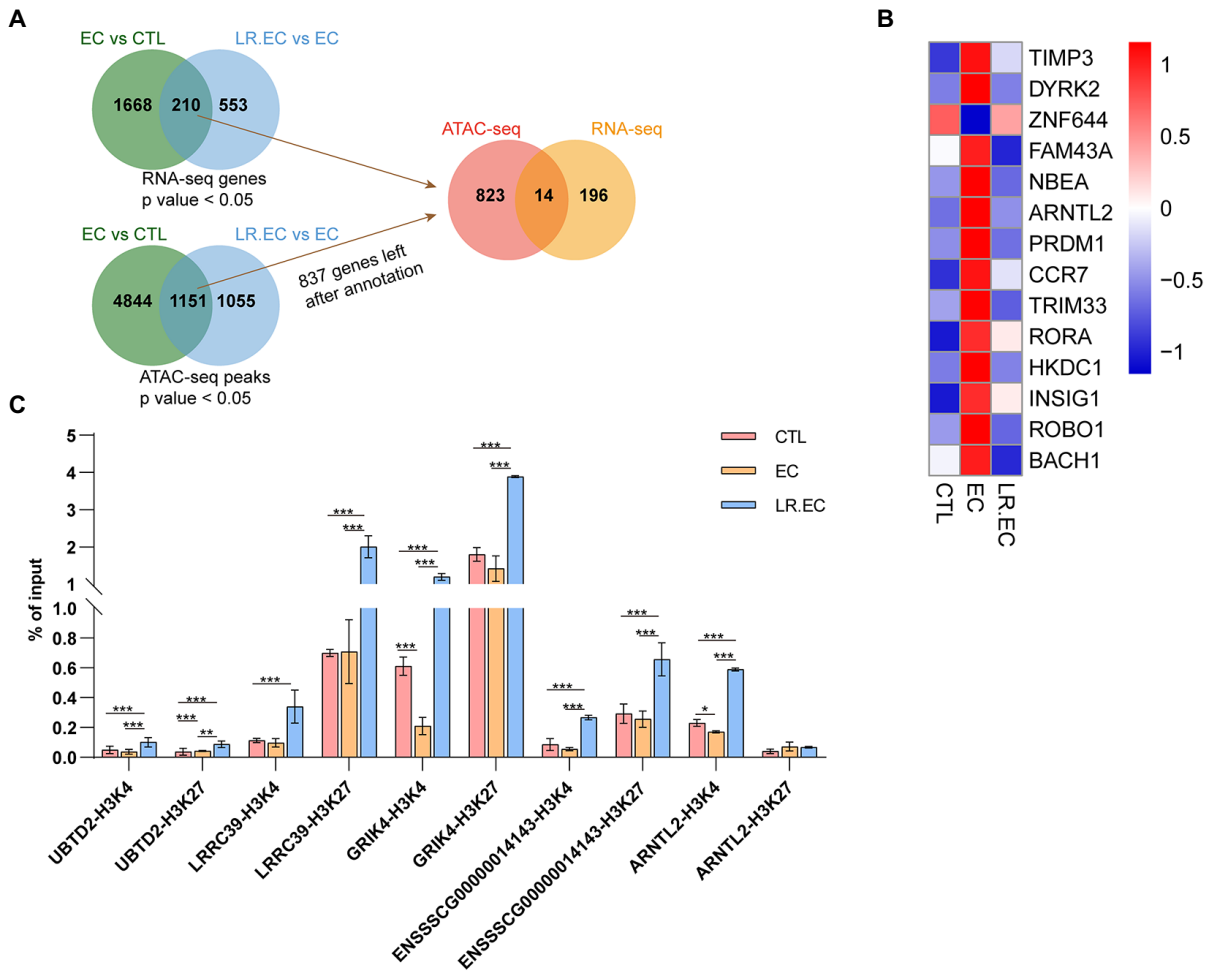
Integrative analysis of RNA-Seq and ATAC-Seq datasets. **(A)** Candidate gene were identified in two steps using overlap: the first step was overlap between different compared groups, and the second step was overlap between DEGs from RNA-seq dataset and annotated genes from ATAC-seq dataset. We set the threshold to a *p* value <0.05; we annotated ATAC-seq peaks to genomic features with the chipseeker package, and only 837 genes had gene names after annotation from DARs. **(B)** Heatmap of 14 overlapping genes in different groups. **(C)** Fold changes of histone H3K4me3 and H3K27ac were determined by ChIP-qPCR in chromatin accessible regions of *UBTD2*, *LRRC39*, *GRIK4*, *ENSSSCG00000014143*, and *ARNTL2*. Data are shown as mean ± SD. *p < 0.05; **p < 0.01; ***p < 0.001.

We detected the accessible chromatin alterations to explore the antibacterial mechanism of *L. reuteri* with ATAC-seq. The results showed that the density distribution of the reads around TSS were strongly enriched (Supplementary Figure S1E), indicating that the chromatin regions were successfully detected by ATAC-seq. We annotated 4,145 genes by the DARs between *E. coli* F18ac and control groups, and 1,481 genes between *E. coli* F18ac with and without *L. reuteri* groups (Supplementary Figures S1F–G). Then, we enriched these genes by GO and KEGG analyses. Genes in the *E. coli* F18a group compared with the control group were most enriched in the tube morphogenesis and the Human papillomavirus infection (Figures 4A,B). Genes in the *E. coli* F18a and *L. reuteri* coincubation group compared with the *E. coli* F18a group were most substantially enriched in the tumor necrosis factor production and the Human papillomavirus infection (Figures 4C,D). We identified pathways such as the PI3K-AKT and MAPK signaling pathways that were also enriched in the RNA-seq analysis; these pathways are closely related to the cell cycle, apoptosis, and cell communication (Engelman et al., 2006; Ertel and Tozeren, 2008), which may be the important regulatory pathways for the antibacterial effect of *L. reuteri*. Furthermore, 14 genes were intersected between the DEGs and the genes annotated by DARs when we set the threshold at *p* < 0.05 (Figure 5A). The visualization of the DEGs’ expression revealed that most of the genes were upregulated after *E. coli* F18ac treatment and downregulated after *L. reuteri*, indicating that *L. reuteri* likely inhibited *E. coli* F18ac by regulating these genes (Figure 5B). However, we noted a low overlap between RNA-seq and ATAC-seq datasets. H3K4me3 and H3K27ac are known to mark active promoters (Calo and Wysocka, 2013), which may lead to changes in chromatin accessibility but do not correspond to differential transcription. Therefore, we investigated the degree of histone H3K4me3 and H3K27ac enrichment of randomly selected genes to explain this phenomenon, and the results showed that H3K4me3 and H3K27ac enrichment increased in regions where chromatin accessibility was not increased, in line with our speculation (Figure 5C).

### 3.4. Regulation of actin cytoskeleton involved in regulation of *Lactobacillus reuteri* mediated bacteriostasis

**Figure 6:**
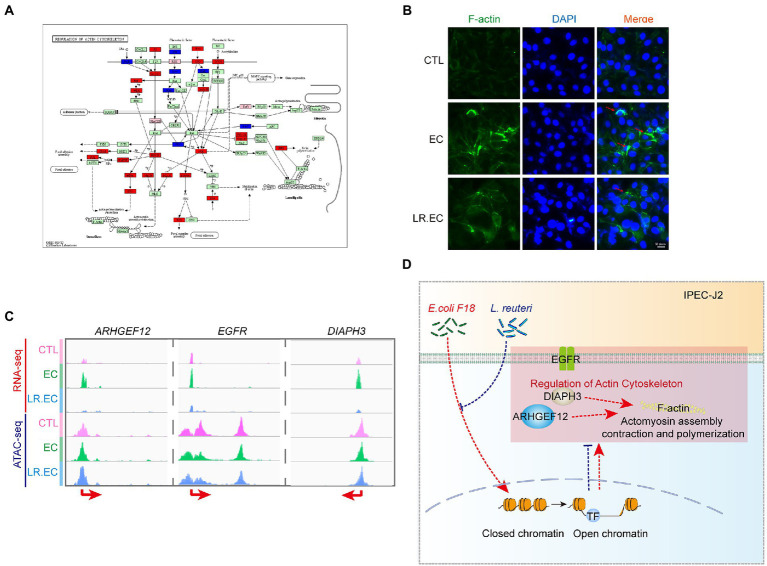
Regulation of actin cytoskeleton involved in regulation of *L. reuteri-*mediated bacteriostasis. **(A)** KEGG map of regulation of actin cytoskeleton. Red indicates genes identified by RNA-seq, blue indicates genes identified by ATAC-seq, and pink indicates genes identified by both. **(B)** IFA assay was used to visualize F-actin in different treatments. The red arrows represent the rearrangement and aggregation of F-actin. **(C)** Visualization of *ARHGEF12, EGFR,* and *DIAPH3* used by IGV in the two datasets. **(D)** Mechanism hypothesis diagram for the regulation of actin cytoskeleton involved in regulation of *L. reuteri-*mediated bacteriostasis. Red dashed lines represent the effect of *E. coli* F18ac, blue dashed lines and crosses represent the effect of *L. reuteri*.

We focused on the regulation of actin cytoskeleton, which was upregulated after *E. coli* F18ac treatment and downregulated after *L. reuteri* treatment in GSEA (Supplementary Figure S2); some peaks of the pathway genes were also notably altered (Figure 6A). We therefore surveyed the effect of different treatments on F-actin, which is the key downstream molecule. We found that considerably rearrangement and aggregation of F-actin occurred after *E. coli* F18ac treatment, which was alleviated by treatment with *L. reuteri* (Figure 6B). Accordingly, we hypothesized that the regulation of the actin cytoskeleton is one of the important regulatory pathways of *L. reuteri* mediated bacteriostasis. We thereafter visualized the genes of the regulation of the actin cytoskeleton both of the datasets, and then identified *ARHGEF12, EGFR,* and *DIAPH3* as potential key genes for which visualization revealed that their expressions were noticeably higher after *E. coli* F18ac treatment. The application of *L. reuteri* restored their expressions (Figure 6C). Enteropathogenic *E. coli* effector EspH can specifically regulate the ARHGEF12 protein, thereby inhibiting the actin cytoskeleton regulated by small G proteins in host cells (Dong et al., 2010). EGFR was identified as a contributor to *E. coli* invasion of the BBB *in vitro* (Wang et al., 2016). Consequently, *ARHGEF12, EGFR,* and *DIAPH3* seem to be critical regulatory molecules in the process of *E. coli* F18ac inhibition by *L. reuteri* (Figure 6D).

## 4. Discussion

*Lactobacillus reuteri*, as a probiotic, can be effectively used in the treatment of diarrhea and other symptoms in children in clinical practice, but we do not have a sufficient understanding of the host regulation mechanism during its antibacterial process. In this study, we examined the transcriptome and chromatin accessibility landscape of IPEC-J2 induced by *E. coli* F18ac and the antibacterial process of *L. reuteri* using RNA-seq and ATAC-seq. To the best of our knowledge, this is the first report of the transcriptome and chromatin accessibility landscape, simultaneously showing differential responses of porcine IPEC-J2 cells to infection with *E. coli F*18ac as well as the inhibition by *L. reuteri*. We confirmed that *L. reuteri* has an inhibitory effect on *E. coli F18ac* through *in vitro* antibacterial experiments. *L. reuteri* can inhibit the growth of *E. coli* and thus inhibit the intestinal infections (Mu et al., 2018). IPEC-J2 cells are reference cellular substrates that can provide useful information about the intestinal interaction between host and enteric pathogens (Mariani et al., 2009). We were able to observe the molecule changes in the cellular responses induced by *E. coli* F18ac and *L. reuteri* with the IPEC-J2 model. We found that many of the *E. coli* F18ac induced DEGs and DARs were enriched in some key signaling pathways, one of which was the PI3K-AKT signaling pathway, which is one of the most important intracellular pathways that regulates cell growth, motility, survival, metabolism, and angiogenesis (Engelman et al., 2006; Alzahrani, 2019). Inhibition of the PI3K-AKT signaling pathway can lead to reduced cell proliferation and increased cell death (Hennessy et al., 2005). Another important pathway was the MAPK pathway, which is located downstream of many growth-factor receptors (Fang and Richardson, 2005) and hence mediates cell communication with extracellular environments (Ertel and Tozeren, 2008). The abundance of DEGs and DARs within the PI3K-AKT and MAPK pathways suggested that these pathways may be involved in *E. coli* F18ac infection and the antibacterial process of *L. reuteri*.

Next, we investigated transcriptional upstream DEGs, and some genes intersected between ATAC-seq and RNA-seq datasets. However, only 33 genes were intersected, which was unexpected. Among them, *TIMP3* and *CCR7* are involved in the regulation of PI3K/AKT pathway activity (Rodriguez-Fernandez and Criado-Garcia, 2020; Yang et al., 2022), and the PI3K/AKT pathway was also identified in our enrichment analysis. These results suggested that *E. coli* F18ac and *L. reuteri* might be involved in the regulation of PI3K/AKT pathway of host cells through regulation expression of *TIMP3* and *CCR7*. *HKDC1* could regulate the AMPK/mTOR signaling pathway to perform its biological function (Wang et al., 2020). *TRIM33* and *BACH1* could regulate the activation of activation of Wnt/β-catenin (Jiang et al., 2015; Li et al., 2022), and *L. reuteri* protect the intestinal mucosal barrier integrity by moderately modulating the Wnt/β-catenin pathway to avoid overactivation (Wu et al., 2020). *DYRK2*, *RORA* and other genes are also involved in the regulation of cell cycle (Lara-Chica et al., 2022), apoptosis (Li et al., 2022) and other activities. The above studies also fully confirmed that these candidate genes identified in this study may be potential regulatory molecules of *L. reuteri* to protect host cells against *E. coli* F18ac. We speculated that one of the reasons why so few genes overlap may be histone modification. Fan, Ren (Fan et al., 2021) also found fewer genes intersected when investigating the association between the specific chromatin-accessible regions and gene expression in murine ovarian GCs upon DON exposure. One potential mechanism is histone acetylation (acetyltransferases/deacetylases) and methylation (methyltransferases/demethylases) between histones and DNA regulating gene expression (Bhaumik et al., 2007). As H3K4me3 and H3K27ac are known to mark active promoters (Calo and Wysocka, 2013), this may lead to changes in chromatin accessibility but does not correspond to differential transcription, hence the lack of overlap in the genes between the two datasets. The ChIP-qPCR results were similar to expectations, and regions with increased chromatin accessibility did not have increased H3K4me3 and H3K27ac enrichment; the trend between the two was inconsistent. Besides, it has been reported that *Lactobacillus mucosae* strain LM1 caused a > 5.1-fold increase in different types of histones, this indicates a significant alteration in protein biosynthesis induced by *L. reuteri* in IPEC-J2 cells (Pajarillo et al., 2017). These both partially explained the reasons for the low overlap. In addition, DNA methylation can also affect gene transcription level. In this study, it was found that demethylated transferase lysine K-specific demethylase (KDM) family showed an overall increasing trend after *E. coli* F18ac infection, while a decreasing trend was showed after the addition of *L. reuteri* (Supplementary Table S5). However, this trend was not significant, and our study lacked data support at the protein level, which needed to be further explored. *L. reuteri* strongly regulates interleukin family members such as IL-1α, IL-6 IL-8, and IL-10 (Ma et al., 2004; Pena et al., 2004; Hoffmann et al., 2008). Therefore, we also investigated the transcriptional changes in interleukin-family genes and found that *E. coli* infection significantly increased the expressions of IL-1α, IL-11, NFIL-3, IL-16, and other cytokines. Zhou et al. identified 867 DEGs (including 30 immune-related genes) in IPECJ2 cells after infection with *E. coli* F18ac (Zhou et al., 2012). The number of DEGs in our study, especially the number of immune-related genes, was lower than that found by Zhou et al., which may have been due to differences in the MOI (we used 1 × 10^7^ CFU/ml in this study, which is lower than the 1 × 10^8^ CFU/ml used by Zhou et al.). Additionally, *L. reuteri* treatment did not reduce the uptrend in the levels of these cytokines, suggesting that porcine *L. reuteri* does not influence *E. coli* F18ac infection in IPEC-J2 cells by modulating interleukin release. The levels of important chemokines CXCL10, and CXCL11 increased only after *L. reuteri* treatment. CXCL10 and CXCL11 regulate immune cell migration, differentiation, and activation (Tokunaga et al., 2018), and the increased levels after *L. reuteri* treatment may have facilitated the recruitment of epithelial cells to immune cells.

Notably, the results of GO and KEGG enrichment analyses did not clearly describe the up- and downregulation changes in DEGs during the bacteriostatic process of *L. reuteri*. Therefore, we performed GSEA, through which we identified 12 pathways that were upregulated after *E. coli* F18ac infection and downregulated after *L. reuteri* treatment; we identified four pathways showing the opposite pattern. Among them, ECM–receptor interaction is involved in cellular–extracellular communication (Cui et al., 2018) and was reported to be associated with the infection process of *E. coli* (Zhou et al., 2012). The actin cytoskeleton can be manipulated by the effector proteins secreted by *E. coli* pathotypes (Selbach and Backert, 2005; Campellone, 2010; Navarro-Garcia et al., 2013). These pathways, which are closely associated with *E. coli* infection, may be a crucial bacteriostatic mechanism of *L. reuteri*. In this regard, we further found that the chromatin accessibility and expression levels of some key genes in the regulation of actin cytoskeleton were altered. The results of the IFA assay revealed that treatment with *E. coli* F18ac markedly augmented actin aggregation, whereas *L. reuteri* treatment effectively lowered this aggregation, indicating that the regulation of the actin cytoskeleton is one of the important mechanisms through which *L. reuteri* inhibits *E. coli* F18ac infection. Additionally, *ARHGEF12* can be specifically regulated by EspH, a type III secretion system effector protein of *E. coli* (Dong et al., 2010), EGFR can also benefit *E. coli* invasion (Wang et al., 2016). Therefore, *ARHGEF12, EGFR,* and *DIAPH3* appear to be the key regulatory targets, which needs to be verified by further experiments.

In summary, our results fully displayed the chromatin accessibility and transcriptional landscape of the *E. coli* F18ac infection processes as well as the bacteriostatic action of *L. reuteri* in IPEC-J2 cells. We identified some key signaling pathways and node genes, revealing that *E. coli* F18ac triggers cellular activities in the host and can be generally regulated by *L. reuteri*. This study provides mechanistic guidance for the use of *L. reuteri* as a probiotic in livestock, poultry, and humans, as well as a theoretical basis for the development and use of *L. reuteri* and its synergistic drugs.

## Data availability statement

The datasets presented in this study can be found in online repositories. The names of the repository/repositories and accession number(s) can be found below: NCBI - PRJNA767941.

## Author contributions

WQ: conceptualization, methodology, and writing–original draft. YX: formal analysis and data curation. ZR: methodology. CX: software. M-aS: software. ZY: resources. WB: funding acquisition and writing–review and editing. All authors contributed to the article and approved the submitted version.

## Funding

This study was supported by grants from the Jiangsu Agricultural Science and Technology Innovation Fund (CX(20)1003), Key Research and Development Project (Modern Agriculture) of Jiangsu Province (BE2019341), and the Priority Academic Program Development of Jiangsu Higher Education Institutions.

## Conflict of interest

The authors declare that the research was conducted in the absence of any commercial or financial relationships that could be construed as a potential conflict of interest.

## Publisher’s note

All claims expressed in this article are solely those of the authors and do not necessarily represent those of their affiliated organizations, or those of the publisher, the editors and the reviewers. Any product that may be evaluated in this article, or claim that may be made by its manufacturer, is not guaranteed or endorsed by the publisher.

## Supplementary material

The Supplementary material for this article can be found online at: https://www.frontiersin.org/articles/10.3389/fmicb.2023.1101111/full#supplementary-material

SUPPLEMENTARY FIGURE S1 Quality metrics and analysis for RNA-seq and ATAC-seq datasets. A. Principal component analysis (PCA) of all RNA-seq samples. B. Wayne diagram of DEGs between *E. coli* F18ac and control groups, C. Wayne diagram of DEGs between *E. coli* F18ac with and without *L. reuteri* groups. B and C, both with a threshold of fold change ≥ 2, adjusted P value < 0.05. D. Principal component analysis (PCA) of all ATAC-seq samples. E. ATAC-seq signal enrichment around 3 kb of the TSS for three representative samples. F. Wayne diagram of genes annotated by DARs between *E. coli* F18ac and control groups, G. Wayne diagram of genes annotated by DARs between *E. coli* F18ac with and without *L. reuteri* groups. F and G, both with a threshold of fold change ≥ 1, *P* value < 0.05.

SUPPLEMENTARY FIGURE S2 Overlapping pathways between different treatments reveal antibacterial process of *L. reuteri*. A. Overlapping pathways that were upregulated after *E. coli* F18ac treatment and downregulated after *L. reuteri* treatment. B. Overlapping pathways that were downregulated after *E. coli* F18ac treatment, and were upregulated after *L. reuteri* treatment.

SUPPLEMENTARY TABLE S1 Details of primers for CHIP-qPCR.

SUPPLEMENTARY TABLE S2 Metadata and mapping statistics for RNA-seq and ATAC-seq.

SUPPLEMENTARY TABLE S3 Pathways enriched by GSEA analysis between E. coli F18ac and control groups.

SUPPLEMENTARY TABLE S4 Pathways enriched by GSEA analysis between E. coli F18ac with and without L.reuteri groups.

## References

[ref1] Alzahrani A. S. (2019). PI3K/Akt/mTOR inhibitors in cancer: at the bench and bedside. Semin. Cancer Biol. 59, 125–132. doi: 10.1016/j.semcancer.2019.07.009, PMID: 31323288

[ref2] Bhaumik S. R. Smith E. Shilatifard A. (2007). Covalent modifications of histones during development and disease pathogenesis. Nat. Struct. Mol. Biol. 14, 1008–1016. doi: 10.1038/nsmb1337 17984963

[ref3] Buenrostro J. D. Giresi P. G. Zaba L. C. Chang H. Y. Greenleaf W. J. (2013). Transposition of native chromatin for fast and sensitive epigenomic profiling of open chromatin, DNA-binding proteins and nucleosome position. Nat. Methods 10, 1213–1218. doi: 10.1038/nmeth.2688, PMID: 24097267 PMC3959825

[ref4] Calo E. Wysocka J. (2013). Modification of enhancer chromatin: what, how, and why? Mol. Cell 49, 825–837. doi: 10.1016/j.molcel.2013.01.038, PMID: 23473601 PMC3857148

[ref5] Campellone K. G. (2010). Cytoskeleton-modulating effectors of enteropathogenic and enterohaemorrhagic *Escherichia coli*: Tir, EspFU and actin pedestal assembly. FEBS J. 277, 2390–2402. doi: 10.1111/j.1742-4658.2010.07653.x, PMID: 20477869

[ref6] Cervantes-Barragan L. Chai J. N. Tianero M. D. Di Luccia B. Ahern P. P. Merriman J.. (2017). *Lactobacillus reuteri* induces gut intraepithelial CD4(+)CD8alphaalpha(+) T cells. Science 357, 806–810. doi: 10.1126/science.aah5825 28775213 PMC5687812

[ref7] Chen L. Zhang Y. H. Zheng M. Y. Huang T. Cai Y. D. (2016). Identification of compound-protein interactions through the analysis of gene ontology, KEGG enrichment for proteins and molecular fragments of compounds. Mol. Gen. Genomics. 291, 2065–2079. doi: 10.1007/s00438-016-1240-x, PMID: 27530612

[ref8] Cockerill F. R. (2010). Performance standards for antimicrobial susceptibility testing twenty-first informational supplement[M]. Clinical and Laboratory Standards Institute, 2010.

[ref9] Cui X. Morales R. T. Qian W. Wang H. Gagner J. P. Dolgalev I.. (2018). Hacking macrophage-associated immunosuppression for regulating glioblastoma angiogenesis. Biomaterials 161, 164–178. doi: 10.1016/j.biomaterials.2018.01.053, PMID: 29421553 PMC8059366

[ref10] DebRoy C. Roberts E. Scheuchenzuber W. Kariyawasam S. Jayarao B. M. (2009). Comparison of genotypes of *Escherichia coli* strains carrying F18ab and F18ac fimbriae from pigs. J. Vet. Diagn. Investig. 21, 359–364. doi: 10.1177/104063870902100310, PMID: 19407090

[ref11] Dias A. M. M. Douhard R. Hermetet F. Regimbeau M. Lopez T. E. Gonzalez D.. (2021). *Lactobacillus* stress protein GroEL prevents colonic inflammation. J. Gastroenterol. 56, 442–455. doi: 10.1007/s00535-021-01774-3, PMID: 33782752

[ref12] Dong N. Liu L. Shao F. (2010). A bacterial effector targets host DH-PH domain RhoGEFs and antagonizes macrophage phagocytosis. EMBO J. 29, 1363–1376. doi: 10.1038/emboj.2010.33, PMID: 20300064 PMC2868573

[ref13] Dore M. P. Bibbò S. Loria M. Salis R. Manca A. Pes G. M.. (2019). Twice-a-day PPI, tetracycline, metronidazole quadruple therapy with Pylera (R) or *Lactobacillus reuteri* for treatment naive or for retreatment of *Helicobacter pylori*. Two randomized pilot studies. Helicobacter 24:e12659. doi: 10.1111/hel.12659, PMID: 31502382

[ref14] Dore M. P. Cuccu M. Pes G. M. Manca A. Graham D. Y. (2014). *Lactobacillus reuteri* in the treatment of helicobacter pylori infection. Intern. Emerg. Med. 9, 649–654. doi: 10.1007/s11739-013-1013-z 24178436

[ref15] Engelman J. A. Luo J. Cantley L. C. (2006). The evolution of phosphatidylinositol 3-kinases as regulators of growth and metabolism. Nat. Rev. Genet. 7, 606–619. doi: 10.1038/nrg1879 16847462

[ref16] Ertel A. Tozeren A. (2008). Switch-like genes populate cell communication pathways and are enriched for extracellular proteins. BMC Genomics 9:3. doi: 10.1186/1471-2164-9-3, PMID: 18177501 PMC2257939

[ref17] Fairbrother J. M. Nadeau E. Gyles C. L. (2005). *Escherichia coli* in postweaning diarrhea in pigs: an update on bacterial types, pathogenesis, and prevention strategies. Anim. Health Res. Rev. 6, 17–39. doi: 10.1079/AHR2005105, PMID: 16164007

[ref18] Fan H. Ren Z. Xu C. Wang H. Wu Z. Rehman Z. U.. (2021). Chromatin accessibility and transcriptomic alterations in murine ovarian granulosa cells upon Deoxynivalenol exposure. Cells 10:2818. doi: 10.3390/cells10112818, PMID: 34831041 PMC8616273

[ref19] Fang J. Y. Richardson B. C. (2005). The MAPK signalling pathways and colorectal cancer. Lancet Oncol. 6, 322–327. doi: 10.1016/S1470-2045(05)70168-6 15863380

[ref20] Grandi F. C. Modi H. Kampman L. Corces M. R. (2022). Chromatin accessibility profiling by ATAC-seq. Nat. Protoc. 17, 1518–1552. doi: 10.1038/s41596-022-00692-9, PMID: 35478247 PMC9189070

[ref21] Hennessy B. T. Smith D. L. Ram P. T. Lu Y. L. Mills G. B. (2005). Exploiting the PI3K/AKT pathway for cancer drug discovery. Nat. Rev. Drug Discov. 4, 988–1004. doi: 10.1038/nrd1902, PMID: 16341064

[ref22] Hoffmann M. Rath E. Holzlwimmer G. Quintanilla-Martinez L. Loach D. Tannock G.. (2008). *Lactobacillus reuteri* 100-23 transiently activates intestinal epithelial cells of mice that have a complex microbiota during early stages of colonization. J. Nutr. 138, 1684–1691. doi: 10.1093/jn/138.9.1684, PMID: 18716170

[ref23] Jiang L. Yin M. Wei X. Liu J. Wang X. Niu C.. (2015). Bach1 represses Wnt/beta-catenin signaling and angiogenesis. Circ. Res. 117, 364–375. doi: 10.1161/CIRCRESAHA.115.306829, PMID: 26123998 PMC4676728

[ref24] Langmead B. Salzberg S. L. (2012). Fast gapped-read alignment with bowtie 2. Nat. Methods 9, 357–359. doi: 10.1038/nmeth.1923, PMID: 22388286 PMC3322381

[ref25] Lara-Chica M. Correa-Saez A. Jimenez-Izquierdo R. Garrido-Rodriguez M. Ponce F. J. Moreno R.. (2022). A novel CDC25A/DYRK2 regulatory switch modulates cell cycle and survival. Cell Death Differ. 29, 105–117. doi: 10.1038/s41418-021-00845-5, PMID: 34363019 PMC8738746

[ref26] Lebeer S. Vanderleyden J. De Keersmaecker S. C. (2008). Genes and molecules of *lactobacilli* supporting probiotic action. Microbiol. Mol. Biol. Rev. 72, 728–764. doi: 10.1128/MMBR.00017-08, PMID: 19052326 PMC2593565

[ref27] Li D. Liu G. Wu Y. (2022). RORA alleviates LPS-induced apoptosis of renal epithelial cells by promoting PGC-1alpha transcription. Clin. Exp. Nephrol. 26, 512–521. doi: 10.1007/s10157-022-02184-2, PMID: 35195816 PMC9114077

[ref28] Li J. Wang X. Su Y. Hu S. Chen J. (2022). TRIM33 modulates inflammation and airway remodeling of PDGF-BB-induced airway smooth-muscle cells by the Wnt/beta-catenin pathway. Int. Arch. Allergy Immunol. 183, 1127–1136. doi: 10.1159/000524574, PMID: 35636393

[ref29] Liu H. B. Hou C. L. Wang G. Jia H. M. Yu H. T. Zeng X. F.. (2017). *Lactobacillus reuteri* I5007 modulates intestinal host defense peptide expression in the model of IPEC-J2 cells and neonatal piglets. Nutrients 9:559. doi: 10.3390/nu9060559, PMID: 28561758 PMC5490538

[ref30] Liu Y. Y. Tian X. J. He B. K. Hoang T. K. Taylor C. M. Blanchard E.. (2019). *Lactobacillus reuteri* DSM 17938 feeding of healthy newborn mice regulates immune responses while modulating gut microbiota and boosting beneficial metabolites. Am. J. Physiol. Gastr. L. 317, G824–G838. doi: 10.1152/ajpgi.00107.2019, PMID: 31482733 PMC6962498

[ref31] Love M. I. Huber W. Anders S. (2014). Moderated estimation of fold change and dispersion for RNA-seq data with DESeq2. Genome Biol. 15:550. doi: 10.1186/s13059-014-0550-8, PMID: 25516281 PMC4302049

[ref32] Ma D. L. Forsythe P. Bienenstock J. (2004). Live *lactobacillus reuteri* is essential for the inhibitory effect on tumor necrosis factor alpha-induced interleukin-8 expression. Infect. Immun. 72, 5308–5314. doi: 10.1128/Iai.72.9.5308-5314.2004, PMID: 15322027 PMC517478

[ref33] Mariani V. Palermo S. Fiorentini S. Lanubile A. Giuffra E. (2009). Gene expression study of two widely used pig intestinal epithelial cell lines: IPEC-J2 and IPI-2I. Vet. Immunol. Immunopathol. 131, 278–284. doi: 10.1016/j.vetimm.2009.04.006, PMID: 19446887

[ref34] Moxley R. A. Duhamel G. E. (1999). Comparative pathology of bacterial enteric diseases of swine. Adv. Exp. Med. Biol. 473, 83–101. doi: 10.1007/978-1-4615-4143-1_7 10659346

[ref35] Mu Q. Tavella V. J. Luo X. M. (2018). Role of *Lactobacillus reuteri* in human health and diseases. Front. Microbiol. 9:757. doi: 10.3389/fmicb.2018.00757, PMID: 29725324 PMC5917019

[ref36] Navarro-Garcia F. Serapio-Palacios A. Ugalde-Silva P. Tapia-Pastrana G. Chavez-Duenas L. (2013). Actin cytoskeleton manipulation by effector proteins secreted by diarrheagenic *Escherichia coli* pathotypes. Biomed. Res. Int. 2013:374395. doi: 10.1155/2013/374395, PMID: 23509714 PMC3591105

[ref37] Ortiz-Rivera Y. Sanchez-Vega R. Gutierrez-Mendez N. Leon-Felix J. Acosta-Muniz C. Sepulveda D. R. (2017). Production of reuterin in a fermented milk product by *Lactobacillus reuteri*: inhibition of pathogens, spoilage microorganisms, and lactic acid bacteria. J. Dairy Sci. 100, 4258–4268. doi: 10.3168/jds.2016-11534, PMID: 28342608

[ref38] Pajarillo E. A. B. Kim S. H. Valeriano V. D. Lee J. Y. Kang D. K. (2017). Proteomic view of the crosstalk between *Lactobacillus mucosae* and intestinal epithelial cells in co-culture revealed by Q Exactive-based quantitative proteomics. Front. Microbiol. 8:2459. doi: 10.3389/fmicb.2017.02459, PMID: 29312173 PMC5732961

[ref39] Pena J. A. Li S. Y. Wilson P. H. Thibodeau S. A. Szary A. J. Versalovic J. (2004). Genotypic and phenotypic studies of murine intestinal *lactobacilli*: species differences in mice with and without colitis. Appl. Environ. Microbiol. 70, 558–568. doi: 10.1128/Aem.70.1.558-568.2004, PMID: 14711688 PMC321283

[ref40] Peterson L. W. Artis D. (2014). Intestinal epithelial cells: regulators of barrier function and immune homeostasis. Nat. Rev. Immunol. 14, 141–153. doi: 10.1038/nri3608 24566914

[ref41] Ramirez F. Dundar F. Diehl S. Gruning B. A. Manke T. (2014). deepTools: a flexible platform for exploring deep-sequencing data. Nucleic Acids Res. 42, W187–W191. doi: 10.1093/nar/gku365, PMID: 24799436 PMC4086134

[ref42] Rodriguez-Fernandez J. L. Criado-Garcia O. (2020). The chemokine receptor CCR7 uses distinct signaling modules with biased functionality to regulate dendritic cells. Front. Immunol. 11:528. doi: 10.3389/fimmu.2020.00528, PMID: 32351499 PMC7174648

[ref43] Selbach M. Backert S. (2005). Cortactin: an Achilles' heel of the actin cytoskeleton targeted by pathogens. Trends Microbiol. 13, 181–189. doi: 10.1016/j.tim.2005.02.007, PMID: 15817388

[ref44] Skjolaas K. A. Burkey T. E. Dritz S. S. Minton J. E. (2007). Effects of *salmonella enterica serovar typhimurium*, or *serovar Choleraesuis*, *lactobacillus reuteri* and *bacillus licheniformis* on chemokine and cytokine expression in the swine jejunal epithelial cell line, IPECJ2. Vet. Immunol. Immunopathol. 115, 299–308. doi: 10.1016/j.vetimm.2006.10.012 17157391

[ref45] Szajewska H. Urbanska M. Chmielewska A. Weizman Z. Shamir R. (2014). Meta-analysis: *Lactobacillus reuteri* strain DSM 17938 (and the original strain ATCC 55730) for treating acute gastroenteritis in children. Benef. Microbes 5, 285–293. doi: 10.3920/BM2013.0056, PMID: 24463209

[ref46] Tokunaga R. Zhang W. Naseem M. Puccini A. Berger M. D. Soni S.. (2018). CXCL9, CXCL10, CXCL11/CXCR3 axis for immune activation - a target for novel cancer therapy. Cancer Treat. Rev. 63, 40–47. doi: 10.1016/j.ctrv.2017.11.007, PMID: 29207310 PMC5801162

[ref47] Wang X. Maruvada R. Morris A. J. Liu J. O. Wolfgang M. J. Baek D. J.. (2016). Sphingosine 1-phosphate activation of EGFR as a novel target for Meningitic *Escherichia coli* penetration of the blood-brain barrier. PLoS Pathog. 12:e1005926. doi: 10.1371/journal.ppat.1005926, PMID: 27711202 PMC5053521

[ref48] Wang X. Shi B. Zhao Y. Lu Q. Fei X. Lu C.. (2020). HKDC1 promotes the tumorigenesis and glycolysis in lung adenocarcinoma via regulating AMPK/mTOR signaling pathway. Cancer Cell Int. 20:450. doi: 10.1186/s12935-020-01539-7, PMID: 32943998 PMC7488676

[ref49] Wu Z. Qin W. Wu S. Zhu G. Bao W. Wu S. (2016). Identification of microRNAs regulating *Escherichia coli* F18 infection in Meishan weaned piglets. Biol. Direct 11:59. doi: 10.1186/s13062-016-0160-3, PMID: 27809935 PMC5093996

[ref50] Wu H. Xie S. Miao J. Li Y. Wang Z. Wang M.. (2020). *Lactobacillus reuteri* maintains intestinal epithelial regeneration and repairs damaged intestinal mucosa. Gut Microbes 11, 997–1014. doi: 10.1080/19490976.2020.1734423, PMID: 32138622 PMC7524370

[ref51] Yang D. Fan L. Song Z. Fang S. Huang M. Chen P. (2022). The KMT1A/TIMP3/PI3K/AKT circuit regulates tumor growth in cervical cancer. Reprod. Biol. 22:100644. doi: 10.1016/j.repbio.2022.100644, PMID: 35661980

[ref52] Yi H. B. Wang L. Xiong Y. X. Wang Z. L. Qiu Y. Q. Wen X. L.. (2018). *Lactobacillus reuteri* LR1 improved expression of genes of tight junction proteins via the MLCK pathway in IPEC-1 cells during infection with Enterotoxigenic *Escherichia coli* K88. Mediat. Inflamm. 20, 1–8. doi: 10.1155/2018/6434910, PMID: 30210262 PMC6120278

[ref53] Yu G. Wang L. G. He Q. Y. (2015). ChIPseeker: an R/Bioconductor package for ChIP peak annotation, comparison and visualization. Bioinformatics 31, 2382–2383. doi: 10.1093/bioinformatics/btv145, PMID: 25765347

[ref54] Zhang Y. Liu T. Meyer C. A. Eeckhoute J. Johnson D. S. Bernstein B. E.. (2008). Model-based analysis of ChIP-Seq (MACS). Genome Biol. 9:R137. doi: 10.1186/gb-2008-9-9-r137, PMID: 18798982 PMC2592715

[ref55] Zheng J. Wittouck S. Salvetti E. Franz C. Harris H. M. B. Mattarelli P.. (2020). A taxonomic note on the genus *lactobacillus*: description of 23 novel genera, emended description of the genus *lactobacillus* Beijerinck 1901, and union of *Lactobacillaceae* and *Leuconostocaceae*. Int. J. Syst. Evol. Microbiol. 70, 2782–2858. doi: 10.1099/ijsem.0.004107, PMID: 32293557

[ref56] Zhou C. Liu Z. Jiang J. Yu Y. Zhang Q. (2012). Differential gene expression profiling of porcine epithelial cells infected with three enterotoxigenic *Escherichia coli* strains. BMC Genomics 13:330. doi: 10.1186/1471-2164-13-330, PMID: 22823589 PMC3472312

