# Supplementary figures and images for: Integrative ATAC-seq and RNA-seq analyses of IPEC-J2 cells reveals porcine transcription and chromatin accessibility changes associated with Escherichia coli F18ac inhibited by Lactobacillus reuteri
Source: Front Microbiol. 2023 Feb 16;14:1101111. doi: 10.3389/fmicb.2023.1101111 (PMC9978113; doi:10.3389/fmicb.2023.1101111)

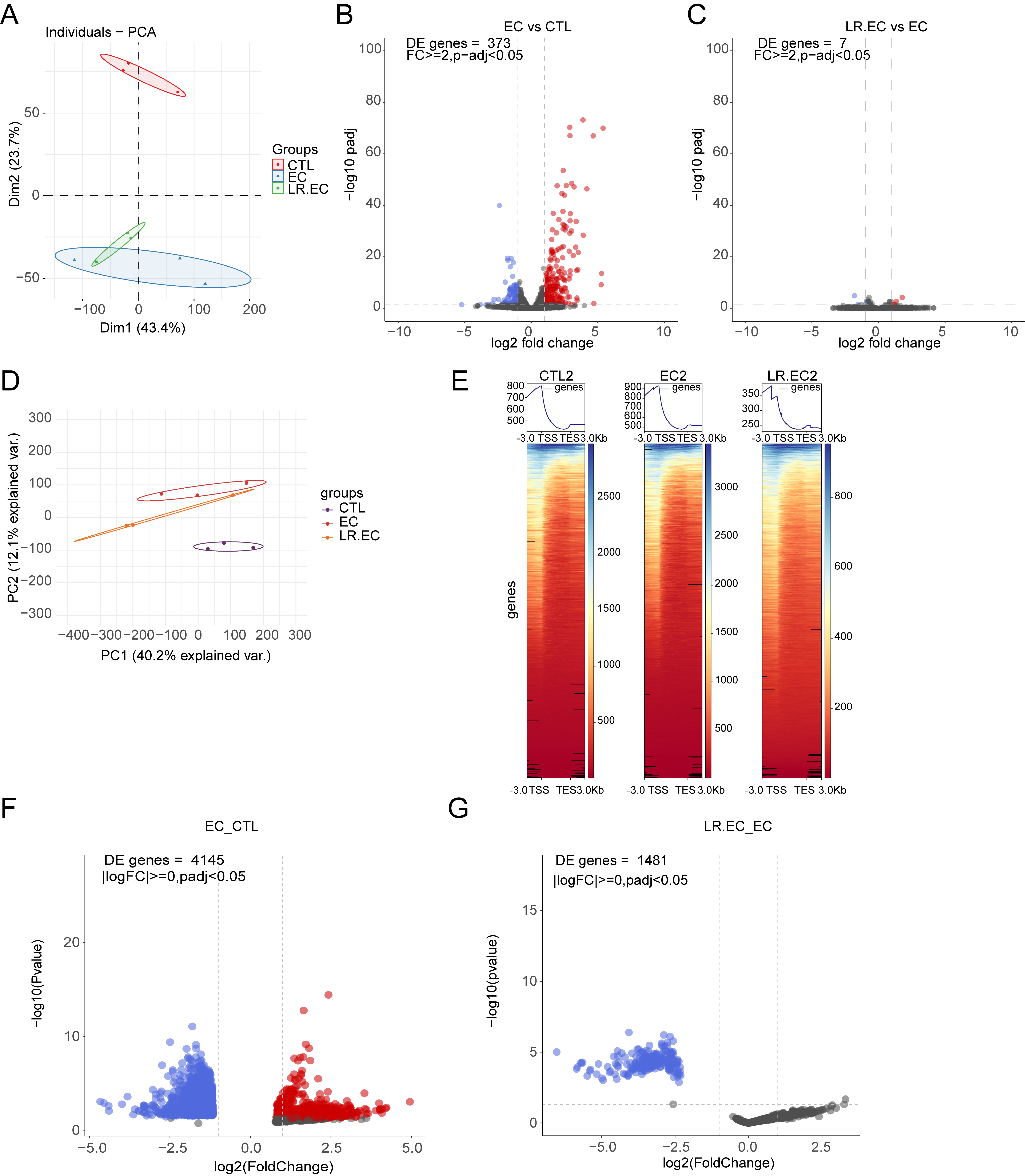

Supplement: SUPPLEMENTARY FIGURE S1 — Quality metrics and analysis for RNA-seq and ATAC-seq datasets. A. Principal component analysis (PCA) of all RNA-seq samples. B. Wayne diagram of DEGs between E. coli F18ac and control groups, C. Wayne diagram of DEGs between E. coli F18ac with and without L. reuteri groups. B and C, both with a threshold of fold change ≥ 2, adjusted P value < 0.05. D. Principal component analysis (PCA) of all ATAC-seq samples. E. ATAC-seq signal enrichment around 3 kb of the TSS for three representative samples. F. Wayne diagram of genes annotated by DARs between E. coli F18ac and control groups, G. Wayne diagram of genes annotated by DARs between E. coli F18ac with and without L. reuteri groups. F and G, both with a threshold of fold change ≥ 1, P value < 0.05. [file Image_1.TIF]

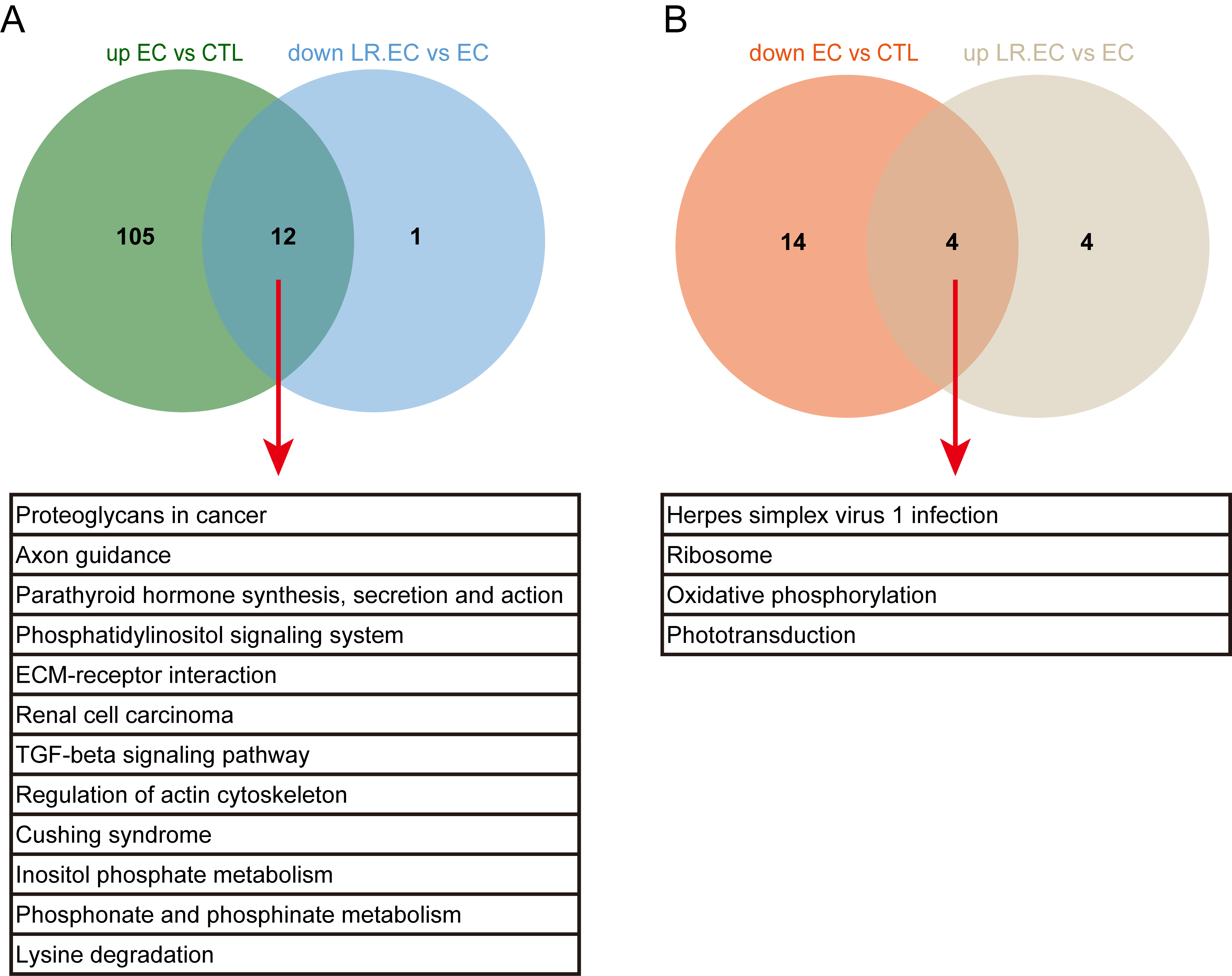

Supplement: SUPPLEMENTARY FIGURE S2 — Overlapping pathways between different treatments reveal antibacterial process of L. reuteri. A. Overlapping pathways that were upregulated after E. coli F18ac treatment and downregulated after L. reuteri treatment. B. Overlapping pathways that were downregulated after E. coli F18ac treatment, and were upregulated after L. reuteri treatment. [file Image_2.TIF]
