# Supplementary material for: Integrative ATAC-seq and RNA-seq analyses of IPEC-J2 cells reveals porcine transcription and chromatin accessibility changes associated with Escherichia coli F18ac inhibited by Lactobacillus reuteri
Source: Front Microbiol. 2023 Feb 16;14:1101111. doi: 10.3389/fmicb.2023.1101111 (PMC9978113; doi:10.3389/fmicb.2023.1101111)
Supplement: SUPPLEMENTARY TABLE S1 — Details of primers for CHIP-qPCR. [file Table_1.DOCX]

**Table S1. Details of primers for CHIP-qPCR**

| **GENE** | **Sequence (5’→3’)** | **Product length (bp)** |
| --- | --- | --- |
| UBTD2 | F: 5'-TTGTGCCGCCTTGAGATA-3' | 286 |
|  | R: 5'-TTGCAGGAAGAATACTGATTTA-3' |  |
| LRRC39 | F: 5'- CTATGCCTCAGTGACGTG-3' | 248 |
|  | R: 5'- TCTGCCAAAGTGGTTATG -3' |  |
| GRIK4 | F: 5'- AGTGCTGCTATGAATGGT-3' | 198 |
|  | R: 5'- GTGTTGGCAAAGATGTGG-3' |  |
| ENSSSCG00000014143 | F: 5'- CCAGCACCTAAACGACTT-3' | 277 |
| ARNTL2 | R: 5'- ACTGGGCCGTATGATAGT -3' |  |
|  | F: 5'- AGATGCTGGAAGGGTGTA -3' | 132 |
